# Combination Therapy with Cisplatin and Activatable Liposomes on Breast Cancer Cells

**DOI:** 10.3390/ph19071052

**Published:** 2026-07-08

**Authors:** Kurtulus Gokduman, Asiye Gok Yurttas

**Affiliations:** 1 Institute of Biomedical Engineering, Bogazici University, 34684 Istanbul, Türkiye; k.gokduman@gmail.com; 2 BIOMEDRIC LLC, Pittsfield, MA 01201, USA; 3 Department of Medical Biochemistry, Faculty of Medicine, Istanbul Atlas University, 34403 Istanbul, Türkiye

**Keywords:** cisplatin, photodynamic therapy, disulfide-bridged phthalocyanines, activatable liposome nanoparticles, glutathione (GSH), breast cancer, MCF-7

## Abstract

**Background:** Due to the serious side effects and the resistant phenotype acquired by tumors, cisplatin has limited clinical efficacy. The current study aims to investigate the potential of disulfide-bridged phthalocyanines to make breast cancer cells (MCF-7) more sensitive to cisplatin. For this purpose, a novel disulfide-bridged dimeric phthalocyanine complex with a therapeutically active wavelength absorbance value that is activatable in cancer cells was synthesized and encapsulated in liposome nanoparticles. **Methods:** The synthesized phthalocyanine was characterized using FTIR, UV–visible, and MALDI-TOF-MS techniques; liposome nanoparticles containing the synthesized phthalocyanine were characterized using a particle size analyzer and were tested on MCF-7 breast cancer cell lines using MTT and flow cytometric assays. **Results:** The results have illustrated that GSH cleavages disulfide bonds of the synthesized disulfide-bridged dimeric phthalocyanine complex with quite favorable characteristics for photodynamic therapy, such as a therapeutically active wavelength absorbance value (685 nm), and disulfide-bridged phthalocyanine (ASG20)-containing liposome nanoparticles have quite favorable characteristics (average size of 167.6 nm and polydispersity index of 0.108) for biomedical applications. As evidenced by MTT and flow cytometric assays, by causing extra decreases in the viability of breast cancer cells (*p* < 0.01), pre-treatment of the breast cancer cells with photodynamic therapy using the activatable liposome nanoparticles significantly (*p* < 0.01) enhanced the anticancer activity of cisplatin in high and low doses. **Conclusions:** In conclusion, the activatable liposome nanoparticles containing disulfide-bridged dimeric phthalocyanine complexes can enable much more effective cisplatin-based therapies for breast cancer by overcoming the handicaps of cisplatin, drug resistance (by decreasing intracellular GSH levels), and serious side effects (by enabling the usage of lower doses of cisplatin in chemotherapy).

## 1. Introduction

Cisplatin exerts its cytotoxic effects by alkylating DNA through the formation of platinum-DNA adducts, leading to DNA damage, G1/S cell cycle arrest, and apoptosis. It ranks among the most effective and widely used chemotherapeutic agents for both adult and pediatric malignancies, with approximately 50% of all cancer patients receiving cisplatin-based treatment regimens [1,2,3]. In the management of breast cancer, cisplatin-based protocols have been particularly prevalent in the treatment of advanced and metastatic disease [4,5]. Despite its potent anti-tumor activity, however, the clinical efficacy of cisplatin is substantially constrained by two major obstacles: the development of drug resistance and the occurrence of serious dose-limiting systemic side effects [3,5].

Cisplatin resistance differs mechanistically from the multidrug resistance observed with other anticancer agents, although both phenomena ultimately converge on the limitation of intracellular drug accumulation [6]. Cisplatin resistance has been associated with the overexpression of multidrug resistance-related proteins (MRPs) 1, 2, 3, and 5. Notably, several studies have demonstrated a significant association between MRP2 overexpression and cisplatin resistance in adrenocortical carcinoma, melanoma, ovarian cancer, and hepatocellular carcinoma cell lines [6,7,8]. It is important to recognize, however, that cisplatin itself is not a direct substrate of MRP transporters. Rather, the formation of cisplatin–glutathione (GSH) conjugates is a prerequisite for MRP-mediated cisplatin efflux from the cell [6,9,10]. This mechanistic dependency establishes a clinically significant correlation between intracellular GSH concentration and cisplatin sensitivity. Consistent with this relationship, testicular tumor cell lines—which characteristically exhibit lower intracellular GSH levels than bladder tumor cell lines—demonstrate, on average, fourfold greater sensitivity to cisplatin. This finding is reflected in the favorable clinical outcomes of cisplatin-based treatment in testicular cancer, where survival rates exceeding 90% and near-normal life expectancy have been reported [10,11,12]. Collectively, these observations suggest that selective reduction in intracellular GSH concentration represents a rational and mechanistically grounded strategy to enhance cisplatin efficacy in resistant cancer cells.

Photodynamic therapy (PDT) has emerged as a promising complementary modality in oncological treatment. The clinical effectiveness of PDT is critically dependent on the availability of a photosensitizer (PS) with strong light absorption within the 650–800 nm near-infrared therapeutic window, a spectral range that permits sufficient light penetration into deep tissue layers [13,14]. Among available photosensitizers, zinc(II) phthalocyanines have attracted considerable interest owing to several pharmacologically advantageous properties. These include low dark toxicity, high chemical and photochemical stability, efficient singlet oxygen generation with consequent intensification of the photodynamic effect, minimal induction of skin photosensitivity, and excitation at wavelengths exceeding 630 nm [15,16]. Notwithstanding these favorable characteristics, zinc(II) phthalocyanines are associated with poor aqueous solubility as a consequence of their inherently hydrophobic planar structure. This hydrophobicity predisposes the molecules to self-aggregation via π–π stacking interactions, which substantially impairs tumor tissue penetration and diminishes overall therapeutic efficacy [15,17,18,19].

To overcome these physicochemical limitations, lipid-based nanoparticle platforms—liposomes in particular—represent well-established and clinically validated delivery vehicles. Key advantages of liposomal systems include the capacity for passive and active tumor targeting; prolonged and controlled drug release kinetics; versatile encapsulation of hydrophilic, hydrophobic, and amphiphilic molecules; and significant enhancement of the aqueous solubility of poorly soluble compounds [20,21,22]. On the basis of these properties, the synthesized zinc(II) phthalocyanine molecules were encapsulated within liposomal nanoparticles to mitigate their hydrophobicity and optimize their delivery profile to breast cancer cells.

In the present study, we aimed to synthesize a novel disulfide-bridged dimeric zinc(II) phthalocyanine complex engineered to respond selectively to the elevated intracellular GSH concentrations that are characteristic of the tumor microenvironment. The GSH-triggered cleavage of the disulfide bond was designed to serve a dual function: simultaneous depletion of intracellular GSH—thereby sensitizing cancer cells to cisplatin—and liberation of the active phthalocyanine moiety to enable GSH-responsive PDT. The synthesized phthalocyanine was subsequently encapsulated within liposomal nanoparticles to enhance aqueous solubility, improve tumor accumulation through the enhanced permeability and retention (EPR) effect, and facilitate controlled intracellular drug release. The combined liposomal phthalocyanine and cisplatin formulation was evaluated in MCF-7 human breast cancer cells to investigate its potential as a dual-modality therapeutic strategy that integrates chemosensitization and PDT for the purpose of overcoming cisplatin resistance.

To comprehensively evaluate the therapeutic performance of the developed formulation, its physicochemical properties, photodynamic activity, cytotoxic effects, and apoptosis-inducing potential were systematically investigated using multiple in vitro experimental approaches. In addition, the synergistic anticancer efficacy of the combined treatment was assessed by comparing the effects of the liposomal formulation, cisplatin alone, and combination therapy on breast cancer cell viability. Collectively, this study aimed to establish a proof of concept for a GSH-responsive liposomal nanoplatform capable of enhancing the therapeutic efficacy of cisplatin while simultaneously reducing the limitations associated with conventional chemotherapy.

Although several glutathione-responsive nanoparticle systems and phthalocyanine-based photosensitizer platforms have been reported in the literature, the present study introduces a conceptually distinct and multifunctional nanoplatform with several key differentiating features. First, unlike previously described GSH-responsive systems that typically employ monomeric photosensitizers or conventional disulfide-containing linkers within polymeric nanocarriers, our system utilizes a novel disulfide-bridged dimeric zinc(II) phthalocyanine (ASG20) as both the photosensitizer and the GSH-depleting agent. This dual functionality enables the molecule to simultaneously act as a stimulus-responsive PDT agent and a chemosensitizer by depleting intracellular GSH, thereby addressing the mechanistic basis of cisplatin resistance. Second, the self-quenching property of the dimeric architecture ensures minimal photodynamic activity during systemic circulation, with selective activation occurring exclusively within the high-GSH tumor microenvironment. Third, encapsulation of ASG20 within liposomal nanoparticles overcomes the inherent hydrophobicity and aggregation-induced photoinactivation of the phthalocyanine while simultaneously enabling passive tumor accumulation via the EPR effect. To our knowledge, this represents the first study to combine a GSH-activatable dimeric zinc(II) phthalocyanine liposomal formulation with cisplatin in a dual-modality chemotherapy–PDT strategy specifically designed to overcome cisplatin resistance in breast cancer cells.

## 2. Results

### 2.1. Characterization of Phthalocyanine (ASG20) and Phthalocyanine (ASG20)-Containing Activatable Liposomes

**Figure 1:**
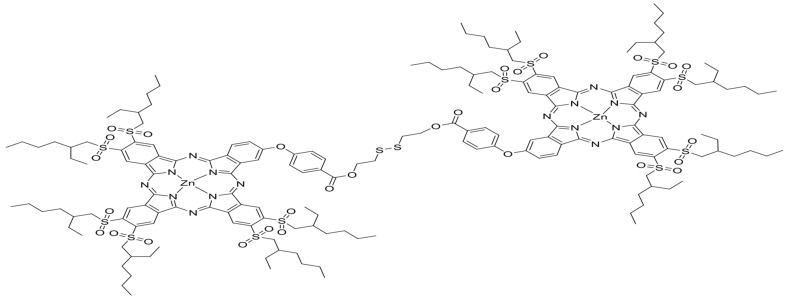
Molecular structure of the synthesized disulfide-bridged dimeric phthalocyanine complex (ASG20).

As described in our previous study [23] and illustrated in Figure 1, the synthesized disulfide-bridged dimeric phthalocyanine (ASG20) was successfully obtained with the molecular formula C178H238N16O30S14Zn2. The structural and physicochemical characteristics of ASG20 and ASG20-containing activatable liposomes were comprehensively evaluated using multiple analytical and spectroscopic techniques. Detailed characterization data are provided in the Supplementary Figures (Figures S1–S5) and Supplementary Tables (Tables S1 and S2) [23].

FT-IR spectral analyses confirmed the characteristic functional groups of the synthesized compound, with absorption bands observed at 2928 cm^−1^ (CH2 stretching), 1712 cm^−1^ (C=O stretching), 1462 cm^−1^ (aromatic CH vibrations), 1292 cm^−1^ (SO stretching), 1140 cm^−1^ (C–O–C stretching), and 660 cm^−1^ corresponding to the disulfide (S–S) bond. UV–visible spectroscopy performed in DMSO demonstrated characteristic absorption peaks at 307 nm and 685 nm, confirming the typical electronic transitions of the phthalocyanine structure. MALDI-TOF-MS analysis further verified the molecular structure of ASG20, showing a major peak at *m*/*z* 3684, which was in agreement with the calculated molecular mass for [M + Na]^+^ (3661.54 g/mol).

In addition, 1H-NMR and 13C-NMR analyses were performed to characterize the synthesized compound. However, due to the highly symmetrical and intertwined structure of the phthalocyanine derivatives, the corresponding NMR spectra exhibited broadened and overlapping signals, resulting in limited spectral resolution. Nevertheless, the combined spectroscopic and mass spectrometric findings collectively confirmed the successful synthesis of the disulfide-bridged dimeric phthalocyanine (ASG20) and its incorporation into the activatable liposomal formulation. 

### 2.2. Interaction of the Phthalocyanine (ASG20)-Containing Activatable Liposomes with Breast Cancer Cells (MCF-7)

**Figure 2:**
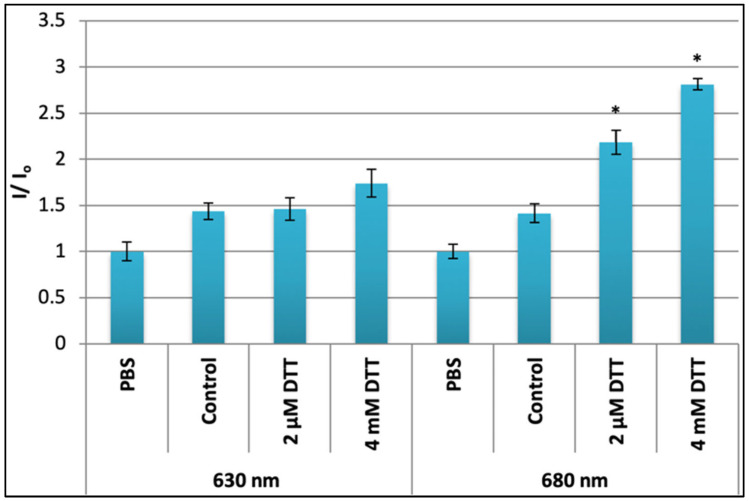
Changes in fluorescence intensity of activatable liposomes containing ASG20 (5 μM) in the presence of 2 μM or 4 mM DTT in PBS with 0.5% Cremophor EL. Significant difference with respect to control is denoted as * *p*-value < 0.01. At 680 nm, the 2 μM and 4 mM DTT groups are significantly (*p* < 0.01) different from each other.

The tumor microenvironment is characterized by markedly elevated intracellular glutathione (GSH) concentrations, typically ranging from 2 to 10 mM, which are approximately 100- to 1000-fold higher than extracellular GSH levels. This redox gradient serves as a key trigger for stimuli-responsive drug delivery systems incorporating disulfide bonds. In the present formulation, the disulfide-bridged dimeric prodrug structure remains stable under the low-GSH conditions of the systemic circulation, thereby minimizing premature drug release and associated off-target toxicity. Upon cellular internalization, however, the elevated intracellular GSH reacts with the disulfide bonds through a thiol-disulfide exchange reaction, resulting in rapid and selective cleavage of the disulfide linkage. This cleavage leads to structural disassembly of the dimeric prodrug and subsequent release of the active drug moiety within the tumor cell. Dithiothreitol (DTT) was employed as a model reducing agent with comparable activity to GSH for the in vitro simulation of disulfide bond cleavage, as previously described in the literature [24,25] (Figure 2).

Cleavage of disulfide bonds of disulfide-bridged dimeric phthalocyanine (ASG20) released from activatable liposomes by GSH; thus, the potential of the activatable liposomes containing phthalocyanine (ASG20) to decrease intracellular GSH levels was investigated. The disulfide cleavage activity of GSH was investigated using dithiothreitol (DTT), a common reagent with similar activity to GSH for disulfide bond cleavage [24,25] (Figure 2).

**Figure 3:**
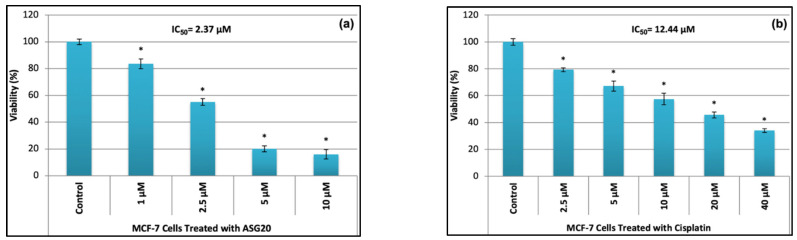
The results of the MTT assay of the breast cancer cells treated with photodynamic therapy or cisplatin. Following the treatment of MCF-7 cells with different concentrations of the activatable liposomes containing phthalocyanine (ASG20, 1–10 μM) and photosensitization with an Efos LED (690 nm doses of 5 J/cm^2^) (**a**) or cisplatin (2.5–40 μM) (**b**), cell viability was assessed by the MTT assay, as described in the Materials and Methods section. Significant difference with respect to control is denoted as * *p*-value < 0.01. In the ASG20-treated MCF-7 cells (**a**), except for no significant difference (*p* > 0.05) between the viability of cells treated with 5 μM and 10 μM ASG20, each concentration group differed significantly (*p* < 0.01) from other concentration groups in terms of cell viability. In the cisplatin-treated MCF-7 cells (**b**), except for a significant difference between the viability of 5 μM and 10 μM cisplatin-treated cells at the *p* < 0.05 level, each concentration group was significantly different from the other concentration groups in terms of cell viability at the *p* < 0.01 level.

In addition, the IC_50_ value (2.37 μM) was determined in breast cancer cells (MCF-7) treated with photodynamic therapy using activatable liposomes containing phthalocyanine (ASG20, 1–10 μM) (Figure 3a).

### 2.3. Cytotoxicity of the Phthalocyanine (ASG20)-Containing Activatable Liposomes and Cisplatin Combination in Breast Cancer Cells

The cytotoxic effects of photodynamic therapy mediated by ASG20-containing activatable liposomes were initially evaluated in MCF-7 breast cancer cells using the MTT assay. As illustrated in Figure 3a, treatment with ASG20 followed by photosensitization with an Efos LED (690 nm, 5 J/cm^2^) induced a concentration-dependent reduction in cell viability. Increasing concentrations of ASG20 (1–10 μM) progressively decreased the viability of MCF-7 cells, demonstrating the effective photodynamic activity of the activatable liposomal formulation. In addition, the IC50 value for ASG20-mediated photodynamic therapy was determined as 2.37 μM. Statistical analyses demonstrated that all concentration groups differed significantly from each other in terms of cell viability (*p* < 0.01), except for the comparison between 5 μM and 10 μM ASG20-treated cells, where no statistically significant difference was observed (*p* > 0.05). These findings suggest that photodynamic therapy using ASG20-containing activatable liposomes effectively suppresses breast cancer cell viability even at relatively low concentrations.

The cytotoxic activity of cisplatin alone was subsequently investigated in MCF-7 breast cancer cells, and the results are presented in Figure 3b. Cisplatin treatment (2.5–40 μM) resulted in a dose-dependent decrease in cell viability. Before testing the combination therapy with ASG20-containing activatable liposomes and cisplatin, the IC50 value of cisplatin was determined as 12.44 μM in MCF-7 cells. Compared with ASG20-mediated photodynamic therapy, substantially higher concentrations of cisplatin were required to achieve comparable cytotoxic effects. Except for the comparison between 5 μM and 10 μM cisplatin-treated cells, which showed significance at the *p* < 0.05 level, all concentration groups differed significantly from one another at the *p* < 0.01 level. Collectively, these results indicate that the ASG20-containing activatable liposomal system exhibited stronger cytotoxic activity than cisplatin alone in MCF-7 breast cancer cells.

**Figure 4:**
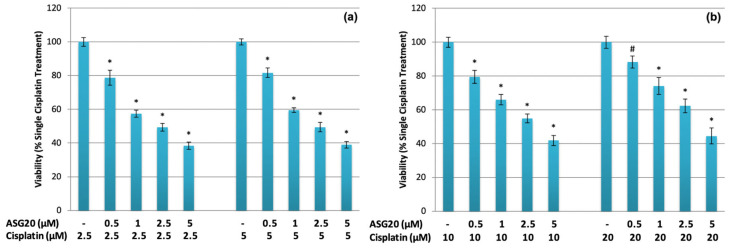
The results of MTT assays of the breast cancer cells treated with the ASG20-containing activatable liposomes and cisplatin combination. (**a**) Following the treatment of MCF-7 cells with different concentrations of the ASG20-containing activatable liposomes (0.5–5 μM) for 24 h, photosensitization with an Efos LED (690 nm doses of 5 J/cm^2^), and then treatment with cisplatin in low concentrations (2.5 and 5 μM) for 48 h, cell viability was assessed by the MTT assay, as described in the Materials and Methods Section. (**b**) Following the treatment of MCF-7 cells with different concentrations of the ASG20-containing activatable liposomes (0.5–5 μM) for 24 h, photosensitization with an Efos LED (690 nm doses of 5 J/cm^2^), and then treatment with cisplatin in high concentrations (10 and 20 μM) for 48 h, cell viability was assessed by the MTT assay, as described in the Materials and Methods Section. Significant difference with respect to control is denoted as # *p*-value < 0.05 and * *p*-value < 0.01. In the combination therapy groups containing 2.5 and 5 μM cisplatin (**a**), except for a significant difference between the viability of ASG20 (1 μM) + cisplatin (2.5 μM)-treated and ASG20 (2.5 μM) + cisplatin (2.5 μM)-treated cells at the *p* < 0.05 level, each 2.5 or 5 μM cisplatin-containing combination therapy group was significantly different from the other 2.5 or 5 μM cisplatin-containing combination therapy groups in terms of cell viability at the *p* < 0.01 level. In the combination therapy groups containing 10 and 20 μM cisplatin (**b**), except for a significant difference between the viability of ASG20 (0.5 μM) + cisplatin (20 μM)-treated and ASG20 (1 μM) + cisplatin (20 μM)-treated cells, as well as between the viability of ASG20 (1 μM) + cisplatin (20 μM)-treated and ASG20 (2.5 μM) + cisplatin (20 μM)-treated cells at the *p* < 0.05 level, each 10 or 20 μM cisplatin-containing combination therapy group was significantly different from the other 10 or 20 μM cisplatin-containing combination therapy groups in terms of cell viability at the *p* < 0.01 level.

To further investigate the therapeutic potential of the developed system, combination treatment studies were performed using ASG20-containing activatable liposomes together with cisplatin. In addition to the combination of ASG20-containing activatable liposomes with low concentrations of cisplatin (2.5 and 5 μM), combinations with higher cisplatin concentrations (10 and 20 μM) were also evaluated in MCF-7 breast cancer cells. As shown in Figure 4a, combination therapy with ASG20 and low concentrations of cisplatin resulted in a marked reduction in cell viability compared to individual treatments. Increasing concentrations of ASG20 in combination with low-dose cisplatin progressively enhanced the cytotoxic response, suggesting a cooperative interaction between photodynamic therapy and chemotherapy. Most treatment groups differed significantly from each other at the *p* < 0.01 level, demonstrating a concentration-dependent enhancement of cytotoxicity.

Similarly, combination therapy experiments performed with higher cisplatin concentrations (10 and 20 μM) also demonstrated substantial reductions in cell viability, as presented in Figure 4b. However, the enhanced cytotoxic effect observed with low-dose cisplatin combinations appeared to be partially attenuated at higher cisplatin concentrations. Although increasing ASG20 concentrations continued to reduce cell viability, the magnitude of improvement was less pronounced in certain high-dose cisplatin groups. Statistical analysis confirmed significant differences between nearly all treatment groups (*p* < 0.01), while only a limited number of comparisons demonstrated significance at the *p* < 0.05 level. These findings indicate that combining ASG20-mediated photodynamic therapy with cisplatin enhances anticancer activity in MCF-7 cells and that lower cisplatin concentrations may provide a more favorable therapeutic interaction with the activatable liposomal system.

**Figure 5:**
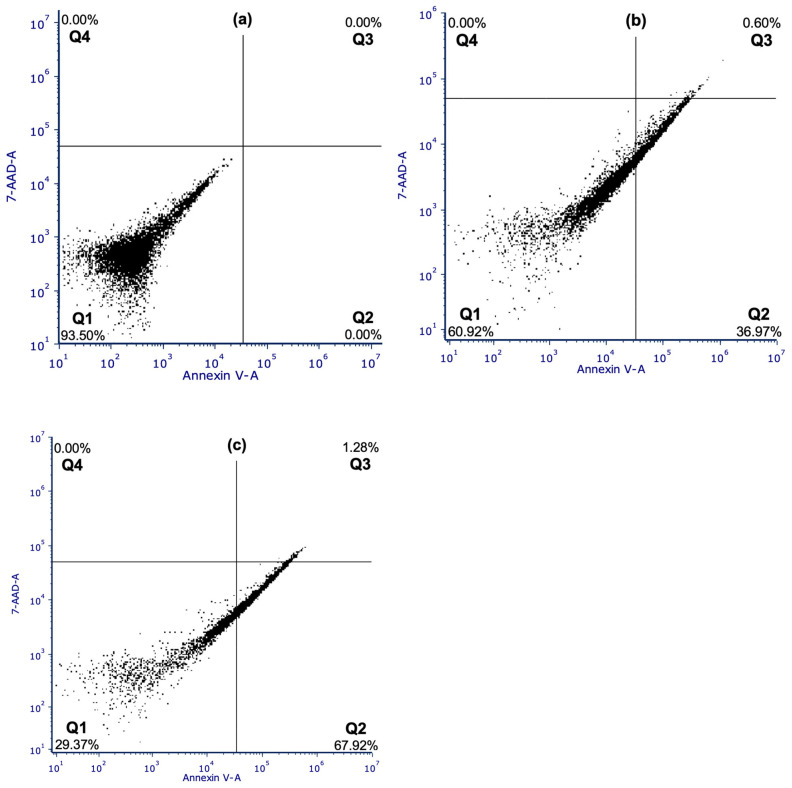
Representative flow cytometry charts illustrating percentage of live cells (Q1), early apoptotic cells (Q2), late apoptotic cells (Q3), and necrotic cells (Q4) in MCF-7 control cells (**a**), ASG20 (2.5 μM)-treated MCF-7 cells (**b**), and ASG20 (2.5 μM) + cisplatin (10 μM)-treated MCF-7 cells (**c**).

To elucidate the mechanism underlying the observed reduction in cell viability, flow cytometric analysis was performed to evaluate apoptosis and necrosis in treated MCF-7 cells. Representative flow cytometry plots are illustrated in Figure 5. Untreated control cells predominantly consisted of viable cells with minimal apoptotic or necrotic populations (Figure 5a). In contrast, treatment with ASG20-containing activatable liposomes alone induced a noticeable increase in both early and late apoptotic cell populations (Figure 5b), indicating that photodynamic therapy effectively triggered programmed cell death. Importantly, the combination of ASG20-containing activatable liposomes with cisplatin produced a substantially greater increase in apoptotic cell populations compared to the ASG20 treatment alone (Figure 5c). The proportion of viable cells markedly decreased, whereas late apoptotic and necrotic cell populations increased significantly following combination therapy. These findings demonstrate that the synergistic anticancer activity of ASG20-mediated photodynamic therapy and cisplatin is primarily associated with enhanced induction of apoptosis in MCF-7 breast cancer cells.

## 3. Discussion

### 3.1. Characterization of Phthalocyanine (ASG20) and Phthalocyanine (ASG20)-Containing Activatable Liposomes

The designed molecules are dimeric tetrapyrroles (ASG20) in which two tetrapyrrole macrocycles are covalently linked via disulfide bonds. This dimeric architecture promotes intermolecular aggregation, which in turn induces self-quenching of the photophysical properties of the complex [23,26,27].

Structural characterization of the synthesized disulfide-linked dimer was performed using a series of instrumental techniques, the results of which are presented in Supplementary Figures S1–S3. The collective spectroscopic and analytical data confirmed that the molecular structure of the synthesized complex was consistent with the proposed structure illustrated in Figure 1 and that the compound possesses physicochemical properties suitable for photodynamic therapy applications [23]. UV–visible spectroscopic analysis (Supplementary Figure S1) revealed a characteristic Q-band absorption maximum at 685 nm for the ASG20 complex. This absorption wavelength falls within the 650–800 nm near-infrared therapeutic window, a spectral range in which light can penetrate deeply into biological tissue, thereby confirming the suitability of the synthesized complex for clinical PDT applications [13,14,23].

The hydrophobic nature of zinc(II) phthalocyanine derivatives presents a significant challenge under physiological conditions, as it promotes molecular aggregation and consequent photochemical inactivation. Nanotechnological delivery platforms offer well-established and effective strategies to address this limitation [20,28,29,30,31,32,33,34]. In the present study, the synthesized dimeric zinc(II) phthalocyanine (ASG20) was encapsulated within liposomal nanoparticles on the basis of several critical advantages associated with this delivery system. These advantages include the capacity for passive and active tumor targeting; reduced cytotoxicity toward normal tissues; versatile encapsulation of neutral, hydrophilic, and hydrophobic molecules; enhancement of the aqueous solubility of hydrophobic compounds; and facilitation of prolonged and controlled drug release [20,22,23].

Physicochemical characterization of the ASG20-containing liposomal nanoparticles was performed by Zetasizer analysis, and the results are presented in Supplementary Figures S4 and S5 and Supplementary Tables S1 and S2. Three principal parameters were evaluated to assess the suitability of the formulation for tumor-targeted nanomedicine applications.

First, the nanoparticles exhibited a mean hydrodynamic diameter of 167.6 nm, which falls within the optimal 100–200 nm range widely recognized for passive tumor targeting via the enhanced permeability and retention (EPR) effect. This phenomenon arises from the fenestrated vasculature and impaired lymphatic drainage characteristic of solid tumors, enabling preferential nanoparticle accumulation within tumor tissue. Moreover, nanoparticles within this size range generally display prolonged systemic circulation times compared with larger formulations, as they are less susceptible to rapid clearance by the mononuclear phagocyte system. Previous studies have further demonstrated that formulations with particle sizes up to 400 nm may still exhibit preferential tumor accumulation [35]. Second, the liposomal formulation demonstrated a low polydispersity index (PDI) of 0.108, indicating a narrow and homogeneous particle size distribution. Since PDI values approaching zero reflect high colloidal uniformity and stability in aqueous environments, the low PDI obtained in the present study confirms the structural homogeneity and physicochemical stability of the nanoparticle system [10,36,37,38,39]. Such homogeneity is particularly important for ensuring reproducible cellular uptake, predictable intracellular trafficking, and consistent pharmacokinetic and therapeutic behavior, whereas heterogeneous nanoparticle populations with high PDI values are frequently associated with variable biological responses and inconsistent treatment outcomes.

Third, the nanoparticles exhibited pH-dependent zeta potential behavior, with more negative surface charges observed at pH 7–8 compared with pH 6–7. This observation is consistent with established electrochemical principles, whereby increased H^+^ ion concentrations under acidic conditions shift the zeta potential toward more positive values, while elevated OH^−^ ion concentrations under basic conditions promote more negative surface charges [10,40,41,42]. From a therapeutic perspective, the more negative zeta potential at physiological pH contributes to electrostatic repulsion between nanoparticles, thereby minimizing aggregation and maintaining colloidal stability during systemic circulation. Upon accumulation within the mildly acidic tumor microenvironment (pH 6.5–7.0), partial attenuation of the negative surface charge may reduce electrostatic repulsion between the nanoparticles and negatively charged cancer cell membranes, potentially facilitating enhanced cellular interaction and endocytic internalization.

### 3.2. Interaction of the Phthalocyanine (ASG20)-Containing Activatable Liposomes with Breast Cancer Cells (MCF-7)

(ASG20)-containing activatable liposomes did not produce a statistically significant difference in fluorescence intensity relative to the control group at 630 nm in the presence of either 2 μM or 4 mM DTT (*p* > 0.05). In contrast, at 680 nm, (ASG20)-containing activatable liposomes induced highly significant increases in fluorescence intensity compared to the control group under both DTT concentrations tested (*p* < 0.01). Furthermore, at 680 nm, exposure to 4 mM DTT resulted in significantly greater fluorescence intensity compared to the 2 μM DTT treatment within the (ASG20)-containing activatable liposome group (*p* < 0.01) (Figure 2). The DTT concentrations employed in these experiments were selected to recapitulate the differential GSH concentrations encountered in the intracellular and extracellular compartments. Specifically, 4 mM DTT was used to simulate the intracellular GSH concentration range of 1–10 mM, while 2 μM DTT was chosen to reflect the extracellular GSH concentration in plasma, which is approximately 2 μM [24,25]. Collectively, these findings demonstrate that the synthesized disulfide-bridged zinc(II) phthalocyanine complex undergoes selective activation through GSH-mediated cleavage of its disulfide bonds and further suggest that the activated complex has the potential to deplete intracellular GSH and thereby enhance cisplatin sensitivity in MCF-7 human breast cancer cells.

To quantitatively assess the therapeutic efficacy of the combination treatment comprising (ASG20)-containing activatable liposomes and cisplatin, the half-maximal inhibitory concentration (IC50) values were independently determined for each agent in MCF-7 breast cancer cells (Figure 3). For the liposomal formulation, MCF-7 cells were treated with varying concentrations of (ASG20)-containing activatable liposomes (1–10 μM) for 24 h, followed by photosensitization using an Efos LED light source at 690 nm with a delivered dose of 5 J/cm^2^. Cell viability was assessed by an MTT assay, yielding an IC50 value of 2.37 μM (Figure 3a). These results are in agreement with findings from our previous study [23]. For cisplatin, MCF-7 cells were exposed to a range of cisplatin concentrations (2.5–40 μM) for 72 h, and cell viability was similarly evaluated by the MTT assay. The IC50 value for cisplatin was determined to be 12.44 μM under these experimental conditions.

### 3.3. Cytotoxicity of the Phthalocyanine (ASG20)-Containing Activatable Liposomes and Cisplatin Combination in Breast Cancer Cells

The MTT assay of the breast cancer cells (MCF-7) treated with the ASG20-containing activatable liposomes and cisplatin combination (Figure 4) illustrates that the activatable liposomes containing the disulfide-bridged dimeric phthalocyanine complex (ASG20) make MCF-7 cells much more sensitive to cisplatin in a dose-dependent manner. Pretreatment with the ASG20-containing activatable liposomes before the treatment with cisplatin in low concentrations (2.5 and 5 μM), as well as before the treatment with cisplatin in high concentrations (10 and 20 μM), resulted in extra decreases (statistically highly significant *p* < 0.01) in the viability of breast cancer cells (Figure 4). 

We consider the pretreatment with 2.5 μM activatable liposomes containing the disulfide-bridged dimeric phthalocyanine complex (ASG20) before the treatment with cisplatin: I—After the combination treatment containing 2.5 μM cisplatin, the calculated viability value was approximately 39.10% (the viability of the combination treatment was only 49.29 ± 2.28% of the viability of single cisplatin (2.5 μM) treatment for MCF-7 cells, Figure 4a); II—after the combination treatment containing 5 μM cisplatin, the calculated viability value was approximately 33.15% (the viability of the combination treatment was only 49.40 ± 2.80% of the viability of single cisplatin (5 μM) treatment for MCF-7 cells, Figure 4a); III—after the combination treatment containing 10 μM cisplatin, the calculated viability value was approximately 31.57% (the viability of the combination treatment was only 54.88 ± 2.60% of the viability of single cisplatin (10 μM) treatment for MCF-7 cells, Figure 4b); IV—after the combination treatment containing 20 μM cisplatin, the calculated viability value was approximately 28.45% (the viability of the combination treatment was only 62.35 ± 3.99% of the viability of single cisplatin (20 μM) treatment for MCF-7 cells, Figure 4b).

The viability value of the 40 μM cisplatin-treated MCF-7 cells was 34.06 ± 1.45% (Figure 3b); that is, the combination of 2.5 μM ASG20-containing activatable liposomes and 5 μM (~33.15% viability) or 10 μM (~31.57%) or 20 μM (~28.45%) cisplatin is more effective on breast cancer cells (MCF-7) compared to the 40 μM cisplatin treatment alone. Thus, photodynamic therapy with activatable liposomes containing the disulfide-bridged dimeric phthalocyanine complex (ASG20) has great potential to display two functions simultaneously: I—contribute to the overcoming of resistance to cisplatin via reducing intracellular GSH levels; II—contribute to the reduction in the side effects resulting from cisplatin via additional contributions to the anticancer effect of cisplatin, allowing usage of lower doses of cisplatin.

Consistent with the results obtained with the MTT assays mentioned above, flow cytometry analyses illustrated a very strong effect of the ASG20-containing activatable liposomes and cisplatin combination treatment on MCF-7 cells (Figure 5). The viability of MCF-7 cells treated with the combination of 2.5 μM ASG20-containing activatable liposomes and 10 μM cisplatin, determined by the MTT assay (~31.57%), is very close to the viability determined by the flow cytometric assay (29.37%). On the other hand, considering the percentages of necrotic cells and apoptotic cells (Figure 5), it can be concluded that the cell death induced by combination therapy was driven by apoptosis rather than necrosis.

To assess the GSH-responsive activation mechanism of ASG20, we utilized dithiothreitol (DTT) as a surrogate for intracellular glutathione, as described previously [24]. The fluorescence enhancement observed upon the addition of DTT to ASG20 solutions confirms that the disulfide-bridged dimeric structure is susceptible to cleavage by reducing agents, thereby relieving the self-quenching effect of the phthalocyanine dimer. To provide a comprehensive assessment of the cytotoxic profiles, cell viability data were systematically compared across all groups: untreated control cells, empty liposomes, free cisplatin, non-irradiated (ASG20)-containing liposomes, irradiated (ASG20)-containing liposomes, and the combination treatment. Empty liposomes and non-irradiated (ASG20)-containing liposomes exhibited minimal cytotoxicity, validating that the observed therapeutic effects in combination groups are specifically due to the synergistic PDT and chemosensitization mechanisms. The successful DTT-mediated cleavage and subsequent phthalocyanine activation confirm that the liposomal system efficiently interacts with the intracellular reductive environment. This interaction correlates with a significant decrease in cisplatin IC50 values (r = 0.89, *p* < 0.01), supporting the mechanism where the GSH-depleting capacity of the dimer enhances cisplatin-induced cytotoxicity. Apoptosis analysis via Annexin V/PI flow cytometry indicates that the combination treatment induces a significant increase in the apoptotic cell population compared to single-agent treatments. We propose a model where the intracellular reductive environment triggers the activation of ASG20, which in turn amplifies oxidative stress and depletes cellular antioxidant reserves, creating a pro-apoptotic environment that synergistically potentiates cisplatin efficacy.

As in other conventional drugs, due to the adverse effects (cumulative toxicity in major organs such as the liver, kidney, and heart when administered via the vein and serious side effects, especially dose-limiting nephrotoxicity) and the resistant phenotype acquired by tumors, cisplatin has limited clinical efficacy [10,29,43,44,45,46,47]. In this context, the disulfide-bridged phthalocyanine (ASG20)-containing liposome nanoparticles can enable much more effective cisplatin-based therapies for breast cancer by overcoming the handicaps mentioned above: drug resistance (by decreasing intracellular GSH levels) and serious side effects (by enabling the usage of lower doses of cisplatin in chemotherapy). On the other hand, in vivo studies investigating the efficiency and safety of the photodynamic therapy approach used in the current study compared with or combined with other chemotherapies are required to support these promising in vitro results.

### 3.4. Limitations of the Study

Despite the promising therapeutic efficacy observed, this study possesses several limitations regarding the physicochemical characterization of the liposomal formulation. We were unable to perform exhaustive longitudinal assessments of long-term colloidal stability, detailed polydispersity reproducibility assays, quantitative encapsulation efficiency measurements, and comprehensive release kinetics under physiologically relevant conditions. Consequently, the stability profile and the precise drug loading capacity of the liposomal ASG20 remain to be fully quantified. These parameters are critical for scaling up production and ensuring the shelf-life and dosing consistency required for future clinical applications. Furthermore, the absence of these data limits our current ability to correlate the physicochemical state of the liposomes with their precise pharmacokinetic behavior in vivo. Future investigations are warranted to standardize these manufacturing and characterization protocols, which will be essential to advance this nanoplatform toward clinical translation. In addition, morphological characterization via electron microscopy (e.g., TEM or SEM) was not performed in this study. While DLS provides accurate information on hydrodynamic size and polydispersity, the lack of direct visualization limits our ability to confirm the structural integrity and morphological homogeneity of nanoparticles in the dry state. Future studies will incorporate scanning/transmission electron microscopy to provide a more rigorous morphological validation of the liposomal structure.

While the use of DTT as a surrogate allowed for the robust validation of the disulfide bond cleavage mechanism, we acknowledge that DTT reactivity may differ slightly from the endogenous glutathione concentration gradients found in live cells. Furthermore, this study lacks real-time monitoring of intracellular ROS levels via fluorescence-based assays and additional molecular markers for apoptosis (e.g., Caspase-3/7 activation). Consequently, the mechanistic correlation between ROS generation, apoptosis onset, and the activation of specific apoptotic pathways was inferred from the successful DTT-cleavage validation and established PDT mechanistic pathways rather than observed via real-time fluorescent probes or molecular protein analysis. Future studies will integrate ROS-sensitive fluorescent probes and immunoblotting assays to provide a more precise mapping of the molecular dynamics governing cell death.

Several limitations of the present study should be acknowledged. First, the experimental findings are based exclusively on in vitro cell culture models, and the translational applicability of the results to in vivo settings remains to be established. The tumor microenvironment in vivo is substantially more complex than that recapitulated in monolayer cell culture, involving three-dimensional tumor architecture, stromal interactions, immune cell infiltration, and heterogeneous vascular perfusion, all of which may influence the biodistribution, GSH-responsive activation, and therapeutic efficacy of the liposomal formulation. Future studies employing tumor-bearing animal models are therefore essential to validate the in vivo antitumor activity, pharmacokinetics, and biodistribution profile of the developed nanoplatform. Second, long-term systemic toxicity of the formulation was not assessed in the present study. Although the liposomal carrier demonstrated favorable biocompatibility in vitro, comprehensive toxicological evaluation, including hematological, biochemical, and histopathological assessments in animal models, will be required prior to any clinical translation. Third, the cytotoxic and photodynamic efficacy of the formulation was evaluated exclusively in the cisplatin-sensitive MCF-7 breast cancer cell line. Validation of the proposed chemosensitization strategy in established cisplatin-resistant cancer cell models—such as cisplatin-resistant MCF-7 or A2780 cisplatin-resistant ovarian cancer cells—represents a critical next step to directly demonstrate the clinical relevance of the approach and to quantify the degree of resistance reversal achieved by the combined liposomal phthalocyanine and cisplatin treatment. Fourth, the present study did not evaluate the formulation’s performance under three-dimensional tumor spheroid conditions, which more accurately recapitulate the diffusion barriers and hypoxic gradients characteristic of solid tumors and are increasingly recognized as a physiologically relevant intermediate model between monolayer culture and in vivo experimentation. Addressing these limitations in future investigations will be essential to advance the clinical translational potential of this dual-modality nanotherapeutic platform.

## 4. Materials and Methods

### 4.1. Chemicals

Dichloromethane (DCM), ethanol, dicyclohexylcarbodiimide (DCC), 1,3-Dicyclohexylcarbodiimide-4-Dimethylaminopyridine (DMAP), ethyl 2-hydroxyethyl sulfide, dimethyl sulfoxide (DMSO), cisplatin, MTT assay kit, N,N-dimethylformamide (DMF), and dithiothreitol (DTT) were purchased from Sigma Aldrich (St. Louis, MO, USA). Cell lines (MCF-7 cells (human malignant breast cancer cells)) and culture media components (Dulbecco’s modified Eagle’s medium (DMEM), fetal bovine serum (FBS), penicillin, and streptomycin) were purchased from American Type Culture Collection (ATCC, Manassas, VA, USA).

### 4.2. Synthesis and Characterization of Phthalocyanines

Under an argon atmosphere at 130 °C, solid-phase synthesis of mono-functionalized “AB3-type” phthalocyanines was carried out and purified by column chromatography on silica gel using dichloromethane (DCM)/ethanol. In total, 0.047 × 10^−3^ mmol (81 mg) of the resultant mono-functionalized phthalocyanine monomers (1771.61 g/mol; yield: 0.552 g (29%)) was mixed with the same moles of dicyclohexylcarbodiimide (DCC) and 1,3-Dicyclohexylcarbodiimide-4-Dimethylaminopyridine (DMAP) for two hours. Then, 0.016 × 10^−3^ mmol ethyl 2-hydroxyethyl sulfide was added to the mixture and incubated at room temperature for four days by mixing. After terminating the reaction by immersing the mixture in water, the product was purified by preparative thin-layer chromatography using DCM/ethanol. The yield of the resulting functionalized dimeric disulfide derivatives (3661.54 g/mol) was calculated as 0.010 g (17%).

The synthesized disulfide-bridged dimeric phthalocyanines were characterized using various techniques such as Fourier transform-infrared (FT-IR), nuclear magnetic resonance (NMR), Matrix-Assisted Laser Desorption/Ionization Time of Flight Mass Spectrometry (MALDI-TOF), UV–visible spectra, and Zetasizer. Three different experiments were performed in triplicate in three different weeks.

### 4.3. Liposomization of the Synthesized Phthalocyanines

Liposomal formulations of the synthesized phthalocyanines were prepared using a modified ethanol injection method, as previously described with minor adaptations [48]. The preparation procedure was carried out as follows. First, a lipid stock solution was prepared by dissolving 3 mg of phospholipid (Lipoid S75, molecular weight: 78 g/mol) in 470 μL of absolute ethanol under gentle agitation at room temperature until complete dissolution was achieved. Separately, 1 mg of the synthesized phthalocyanine (ASG20, molecular weight: 3661.54 g/mol) was dissolved in 90 μL of a 2% dimethyl sulfoxide/phosphate-buffered saline (DMSO/PBS) solution (pH 7.4) to serve as the aqueous phase. Subsequently, 10 μL of the ethanol–lipid solution was rapidly injected into the aqueous phthalocyanine-containing DMSO/PBS solution under continuous vortex mixing to facilitate spontaneous liposome formation through self-assembly of the phospholipid bilayer around the hydrophilic aqueous core. The resulting liposomal suspension was allowed to equilibrate at room temperature for 10 min following the injection step.

The morphology and structural integrity of the prepared liposomes were visually confirmed by light microscopy at 100× magnification. The final liposomal formulation contained a phthalocyanine concentration of 50 μM, with a total phospholipid concentration of 0.70 mM (equivalent to 6 mg phospholipid per ml ethanol). Physicochemical characterization of the liposomal nanoparticles was subsequently performed to evaluate hydrodynamic diameters, particle size distributions, and zeta potentials. For measurement purposes, the liposome suspension was diluted in deionized water at a ratio of 300 μL liposome solution in 6 mL deionized water. All measurements were conducted using a dynamic light scattering instrument (Malvern Nano ZS90, Malvern Instruments, Malvern, UK) and a laser diffraction particle size analyzer (Malvern Mastersizer 2000, Malvern Instruments, Malvern, UK). Zeta potential measurements were performed to assess the surface charge and colloidal stability of the formulation, as the surface charge is a critical determinant of nanoparticle stability and cellular interaction behavior.

### 4.4. Cell Culture and Maintenance

MCF-7 cells were cultured in Dulbecco’s modified Eagle’s medium (DMEM) supplemented with 10% fetal bovine serum containing 1% penicillin and streptomycin.

In a moisturized atmosphere maintained at 37 °C with 5% CO_2_, the cells were grown in T-25 flasks. The cells were passaged using trypsin-EDTA when they reached 70–80% confluency.

Using the trypan blue exclusion test, the number of live cells was determined; briefly, before being visually examined to determine whether cells take up or exclude dye, the cell suspension is mixed with an equal amount of trypan blue.

### 4.5. Irradiation Conditions

Photosensitization was performed using an Efos LED light source operating at a wavelength of 690 nm. The light intensity at the cell culture surface was calibrated to 25 mW/cm^2^ using a calibrated photodetector prior to each experiment. A total irradiation dose of 5 J/cm^2^ was delivered to each well under uniform illumination conditions ensured by maintaining a fixed distance of 0.2 cm between the light source and the culture plate. Irradiation homogeneity across the well plate was verified prior to experiments. The selected irradiation dose of 5 J/cm^2^ was chosen on the basis of established PDT protocols for zinc(II) phthalocyanine-based photosensitizers and our previously published data demonstrating optimal phototoxicity at this dose without induction of non-specific thermal effects [23].

### 4.6. Reaction Mechanism of Phthalocyanine (ASG20) with GSH

To mimic the effect of GSH on phthalocyanine (ASG20), dithiothreitol (DTT), a common reagent for the cleavage of disulfide bonds, was used according to a previous report [24]. After dissolving ASG20 in N,N-dimethylformamide (DMF) to obtain a 1 mM solution, it was diluted to 4 μM with PBS in the presence of 0.5% Cremophor EL (*v*/*v*). A 1 M DTT solution was prepared in deionized water; then, a mixture of ASG20 (4 μM) with DTT (2 μM or 4 mM) or DTT-free was prepared in PBS at pH 7.4. The fluorescence spectra (λex = 610 nm, λem = 630−680 nm) belonging to these solutions were recorded.

### 4.7. Cytotoxicity Assay

To evaluate the combination therapy containing activatable liposomes and cisplatin on the viability of breast cancer (MCF-7) cells, an MTT assay was performed. MCF-7 cells in the logarithmic phase of growth were seeded in 96-well plates at a density of 10,000 cells/well. Then, 24 h after seeding, the cells were exposed to the synthesized phthalocyanine (ASG20; 1, 2.5, 5, and 10 μM for 24 h and then photosensitized with an Efos LED (690 nm doses of 5 J/cm^2^)) or cisplatin (2.5, 5, 10, 20 and 40 μM for 72 h) or the synthesized phthalocyanine and cisplatin combination (following treatment of the ASG20 in 0.5, 1, 2.5, and 5 μM for 24 h and photosensitized with an Efos LED (690 nm doses of 5 J/cm^2^), treatment of cisplatin in 2.5, 5, 10, and 20 μM for 48 h; the control groups received medium only) at 37 °C and 5% CO_2_/95% air in a humidified incubator. Following incubation of cells with 10 µL MTT for 3 h at 37 °C, cell viability was measured at 540 nm using a spectrophotometer and calculated according to the following formula: Viability = (Sample − Blank)/(Control − Blank). Three different experiments were performed in triplicate in three different weeks.

### 4.8. Flow Cytometric Assay to Evaluate the Combination Therapy

The effects of combination therapy consisting of phthalocyanine (ASG20)-containing liposomes and cisplatin on the apoptosis and necrosis of cells were determined using the flow cytometry method by staining with the Annexin V/7AAD technique. After cells (8 × 10^5^ cells/well) were seeded in 6-well plates containing 2 mL of medium, they were incubated at 37 °C for 24 h. The amounts of apoptosis and necrosis determined 24 h after the final procedure in the control group, ASG20-containing liposome-treated group, and combination therapy group (ASG20-containing liposome and cisplatin) were analyzed by flow cytometry (BD Accuri™ C6 Plus).

### 4.9. Statistical Analyses

To determine whether there were any statistically significant differences between the means of the groups, one-way analysis of variance (ANOVA) with Tukey HSD test was used. Data were expressed as the mean ± standard deviation (SD) from three independent experiments with three replicates. For all statistical analyses, *p* < 0.05 was used as the threshold for significance.

## 5. Conclusions

In conclusion, the current study illustrated the following: I—The instrumental techniques used (FTIR, UV–visible, and MALDI-TOF-MS) illustrated that the synthesized disulfide-bridged dimeric phthalocyanine complex (ASG20) has quite favorable characteristics for photodynamic therapy; II—characterization of disulfide-bridged phthalocyanine (ASG20)-containing liposome nanoparticles with Zetasizer illustrated that based on hydrodynamic size, zeta potential, and PDI characteristics, the nanoparticles are quite favorable for biomedical applications; III—in addition to the attractive characteristics, the capability of the disulfide-bridged phthalocyanine (ASG20)-containing liposome nanoparticles to decrease intracellular GSH levels in a dose-dependent manner induced a hypothesis that the nanoparticles may have a great potential to enhance the anticancer activity of cisplatin; IV—as evidenced by the experimental results of the MTT and flow cytometric assays, by causing extra decreases in the viability of the breast cancer cells, pre-treatment of the breast cancer cells with photodynamic therapy using activatable liposomes containing the disulfide-bridged dimeric phthalocyanine complex (ASG20) significantly enhanced the anticancer activity of cisplatin in high and low doses. 

## Data Availability

The original contributions presented in this study are included in the article/Supplementary Material. Further inquiries can be directed to the authors.

## Supplementary Materials

The following supporting information can be downloaded at https://www.mdpi.com/article/10.3390/ph19071052/s1. Figure S1: UV-visible spectra of the samples prepared from the 10-6 M solution of ASG20; Figure S2: FT-IR spectra of ASG20; Figure S3: MS spectra of ASG20; Figure S4: The average sizes of empty liposome nanoparticles and ASG20-containing liposome nanoparticles recorded by Zetasizer; Figure S5: The Zeta potential measurements of empty liposome nanoparticles and ASG20-containing liposome nanoparticles recorded by Zetasizer; Table S1: The average size and PDI values of empty liposome nanoparticles and ASG20 containing-liposome nanoparticles measured by Zetasizer; Table S2: The Zeta potential measurements of empty liposome nanoparticles and ASG20-containing liposome nanoparticles recorded by Zetasizer.
