# Supplementary material for: Combination Therapy with Cisplatin and Activatable Liposomes on Breast Cancer Cells
Source: Pharmaceuticals (Basel). 2026 Jul 8;19(7):1052. doi: 10.3390/ph19071052 (PMC13414501; doi:10.3390/ph19071052)
Supplement: Supplementary file 1 [file pharmaceuticals-19-01052-s001.zip › pharmaceuticals-4314490-supplementary.pdf]

SUPPLEMENTARY INFORMATION

Combination Therapy with Cisplatin and Activatable Liposomes on Breast Cancer Cells#

Kurtulus Gokduman [1] and Asiye Gok Yurttas [2,3,*]

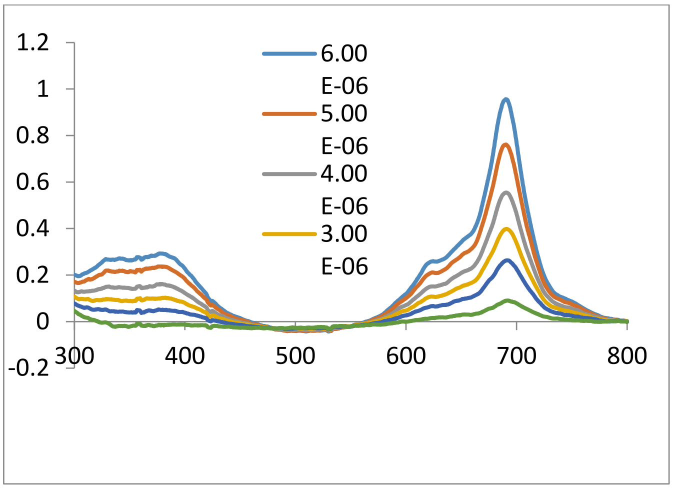

Figure S1. UV-visible spectra of the samples prepared from the 10^{-6} M solution of ASG20.

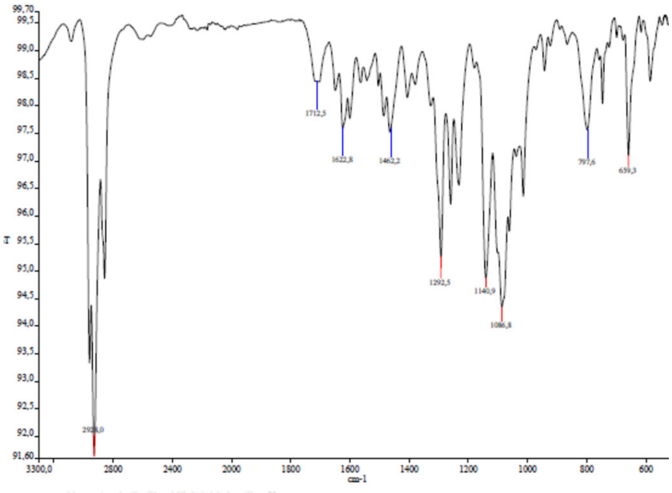

Figure S2. FT-IR spectra of ASG20.

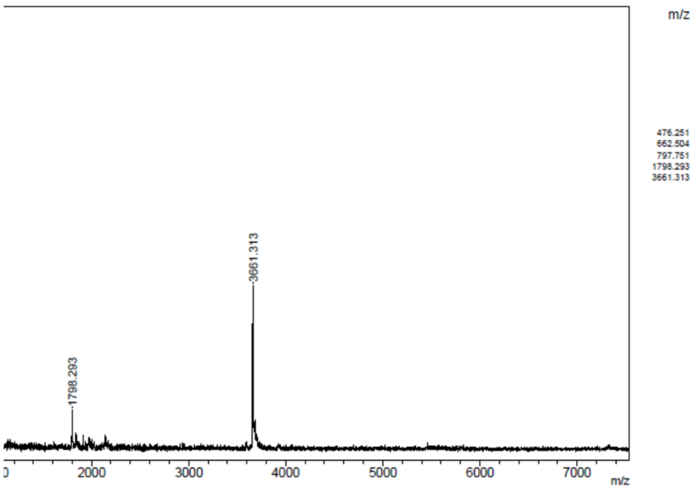

Figure S3. MS spectra of ASG20.

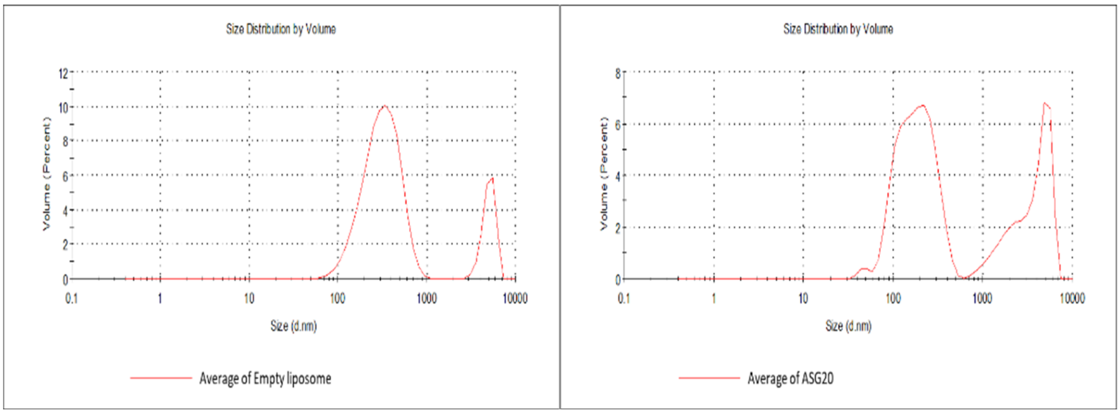

Figure S4. The average sizes of empty liposome nanoparticles, and ASG20-containing liposome nanoparticles recorded by Zetasizer.

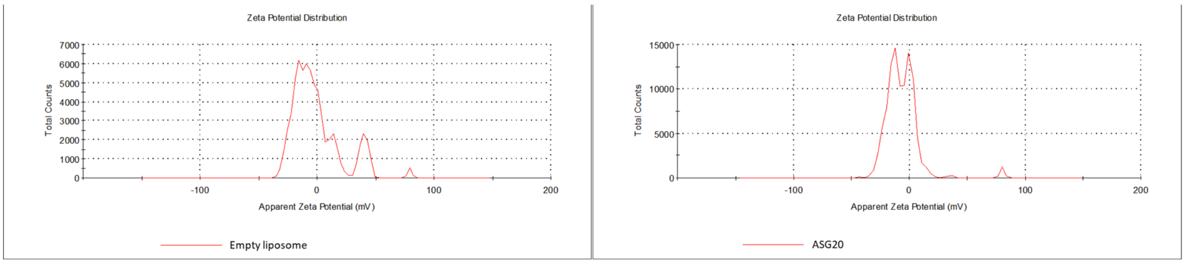

Figure S5. The Zeta potential measurements of empty liposome nanoparticles and ASG20-containing liposome nanoparticles recorded by Zetasizer.

Table S1. The average size and PDI values of empty liposome nanoparticles and ASG20 containing-liposome nanoparticles measured by Zetasizer.

	**Average particle size (nm)**	**Polydispersity index (PDI)**
Empty Liposome	226,4	0,149
ASG20-containing Lipsome	167,6	0,108

Table S2. The Zeta potential measurements of empty liposome nanoparticles and ASG20-containing liposome nanoparticles recorded by Zetasizer.

	Zeta Potential (mV) (pH 6-7)	Zeta Potential (mV) (pH 7-8)
Empty Liposome	-1,15	-1,62
ASG20-containing Lipsome	-6,36	-7,12
